# Real-time measurement of fibers using an HY-differential mobility analyzer with an optical particle counter (KOFAM)

**DOI:** 10.1371/journal.pone.0182119

**Published:** 2017-08-09

**Authors:** Sungwon Choi, Kwangmyung Jang, Kyunghoon Park, Hyunwook Kim

**Affiliations:** 1 Occupational Lung Disease Institute, Korea Workers’ Compensation and Welfare Service, Incheon, Republic of Korea; 2 Department of Preventive Medicine, College of Medicine, The Catholic University of Korea, Seoul, Republic of Korea; Institute for Bioscience and Biotechnology Research, ITALY

## Abstract

This study investigated the applicability of an HY-differential mobility analyzer with an optical particle counter (HY-DMA/OPC), named as KOFAM, for counting fibrous matters in real time. Fibers separated from particles by the HY-DMA were counted with an OPC. To assess the KOFAM performance, the proposed method and the conventional gold standard phase contrast microscopy (PCM) method were compared in terms of variables such as recovery, relative difference, coefficient of determination, and conformity. The optimal sheath-to-aerosol (outlet) flow ratio of the internal flow in the HY-DMA was determined to be 1.6:1. In terms of recovery of the HY-DMA, the highest recovery was obtained at a voltage of 500 V regardless of which type of asbestos was tested. The recovery rate for serpentine was 45.5% and that for amphibole was 34.9%. The coefficients of determination of serpentine (R^2^ = 0.89) and amphibole (R^2^ = 0.87) were highly correlated. With respect to the coefficient of variation (CV), the KOFAM demonstrated superior performance over the M7400AD and F-1 methods and showed almost no difference from the PCM method (KOFAM: 22.5%, M7400AD: 32.4%, F-1: 88.8%, and PCM: 21.9%). There was no statistically significant difference between concentration measurements of the KOFAM and PCM analyses. Accordingly, it was concluded that the KOFAM can be used as a superior alternative to conventional fiber measurement methods. The preliminary results support the use of the KOFAM for constant measurement of airborne asbestos concentrations in real time.

## Introduction

Asbestos, referring to all fibrous natural silicate minerals, is a natural mineral from the serpentine and amphibole mineral families transformed to fibers. It offers excellent durability, heat resistance, chemical resistance, and electrical insulation. Being readily available at low cost, asbestos has been used in a wide range of areas in construction, consumer electronics, and home appliances. If it enters the body, asbestos may cause lung cancer or malignant mesothelioma and asbestos-related diseases such as asbestosis after a long incubation period. For this reason, the International Agency for Research on Cancer (IARC) of the World Health Organization (WHO) has classified it as “carcinogenic to humans (Group 1)” [1].

Asbestos is usually scattered in regions with naturally occurring asbestos (NOA) or in buildings that make use of asbestos-containing materials (ACMs). NOA regions are areas where asbestos is naturally included in rocks or where the soil contains asbestos due to the influence of weathering. These asbestos-containing regions are used as farmland, agricultural complexes, or residential areas, and residents are found likely to potentially develop cancer due to their exposure to asbestos. Therefore, local residents in the NOA regions need to be protected from asbestos exposure from the surrounding atmosphere [2].

As buildings that make use of ACMs gradually deteriorate, there is growing concern about asbestos scattering from buildings to the atmosphere. Consequently, the number of asbestos demolition and removal projects is steadily increasing. In particular, demolition, expansion, or restoration work of facilities or buildings built with ACMs in urban areas with a high population density could scatter asbestos in the atmosphere, exposing workers and the surroundings to it. Therefore, special attention should be paid to consistently measuring the asbestos concentration level in the air during the work [3].

Measurement of asbestos in air is achieved by passing a known volume of air through a filter followed by subsequent treatments of the filter and phase contrast microscopy (PCM) [4]. However, PCM has certain drawbacks in that it requires considerable manpower and time to perform the analysis and is difficult to verify the results in real time. The ability to measure asbestos fibers in real time has become a need in various fields. Currently, there are four real-time measurement devices that are commercially available, namely M7400AD (MSP Corp., USA), Fibrecheck (Casella, UK), ALERT (Select Group, UK), and F-1 fiber monitor (Sibata, Japan). However, even these instruments are not yet competent enough to replace PCM in terms of accuracy with regard to asbestos measurement. Therefore, there is a need for a field instrument capable of accurately measuring asbestos in real time. To achieve this goal, in this study, a differential mobility analyzer (DMA) was investigated as an alternative to existing equipment.

For the assessment of nano-sized fibers, DMA-based technology is considered a reliable means of measuring fibrous matter in real time. A DMA is the most commonly used device for classifying aerosol nano-particles by size, and is based on electrical mobility [5]. In the measurement process, carbon nano-tubes (CNTs) or nano-fiber materials pass through the DMA. After being separated according to differences in electrical mobility, they are counted by a condensation nucleus/particle counter (CNC/CPC). Multiple studies have been conducted using a DMA to measure nano-fiber materials. To understand the dimensional characteristics of CNTs and nano-fibers, fibrous matter must be separated according to varied electrical mobility by applying voltage within the DMA, drawing on the principle of electrophoresis [6]. Previously, ultra-microscopic fibrous CNTs were successfully separated using a DMA and their characteristics were examined with regard to voltage stress [7]. CNTs have also been effectively sifted based on the principle of different electrical mobility for different types of fibrous matter under a certain voltage within the DMA [8,9]. Using a DMA, Kim et al. (2009) separated fibrous matter from chemical vapor deposition for the purposes of particle size classification and other analysis [10]. In another study, a modified DMA shorter than conventional DMAs was employed for nano-particle separation. It was confirmed that nano-particles comprising molecular ions could be also separated in an electrospray state using the modified DMA [11]. As such, reliable extraction of nano-sized fibrous matter has been verified through several studies.

However, no study has investigated applying DMAs to micro-level fibrous matter, except for one study in which the charge number of asbestos obtained through unipolar diffusion was lower than the theoretically calculated number by 5–10% [12]. There was also an attempt to develop a method to separate fibrous matter by increasing the length of existing DMAs, but it was not practically feasible because the size of the DMA became too big. Currently, there are not many ways to effectively separate fibrous matter from particles. Recently, a new device, called an HY-DMA, was developed. Instead of charging particles like a traditional DMA does, this technology first electro-neutralizes particles in the air to convert them to an electro-statically neutral state. Aerosols then pass through a non-uniform electric field to form a dipole moment around the fiber-shape particles within the HY-DMA. The device then uses mobility differences to separate fibrous matter from particulate [13]. This study employed an HY-DMA to separate fibers and measured the separated fibers in real time using an optical particle counter (OPC). Previously, there have been cases in which fibrous matter was measured with OPC. Rickards (1978) used standard OPC to detect the concentration of asbestos in work places where the majority of surrounding aerosols comprised fibrous matter [14]. Sachweh et al. (1999) analyzed the correlation between the size and concentration of fibers by measuring 12 μm-long SiO_2_ fibers with OPC [15]. Thus, objective of this study was to test the applicability of an HY-DMA used in tandem with an OPC for measuring fibrous matter in real time. It was named as the Korea Fibrous Aerosol Monitor, KOFAM.

## Materials and methods

### 1. Preparation of samples

Two different types of asbestos samples, serpentines (chrysotile) and amphiboles (amosite, tremolite, and actinolite) were purchased from RTI International, USA, for use in the experiment. The asbestos samples were prepared by pulverizing for five minutes [16] and mixing with sand. They were passed through a 60 mesh (250 μm) standard sieve at fixed ratios, and mixtures with an asbestos content of 1 × 10−5–0.1% (w/w) were generated. The weight was measured up to the fifth decimal digit (10^−5^) using a balance (Ohaus, USA), and the weighed samples were homogenously mixed with a vortex mixer.

### 2. Fiber generation and sampling

**Fig 1:**
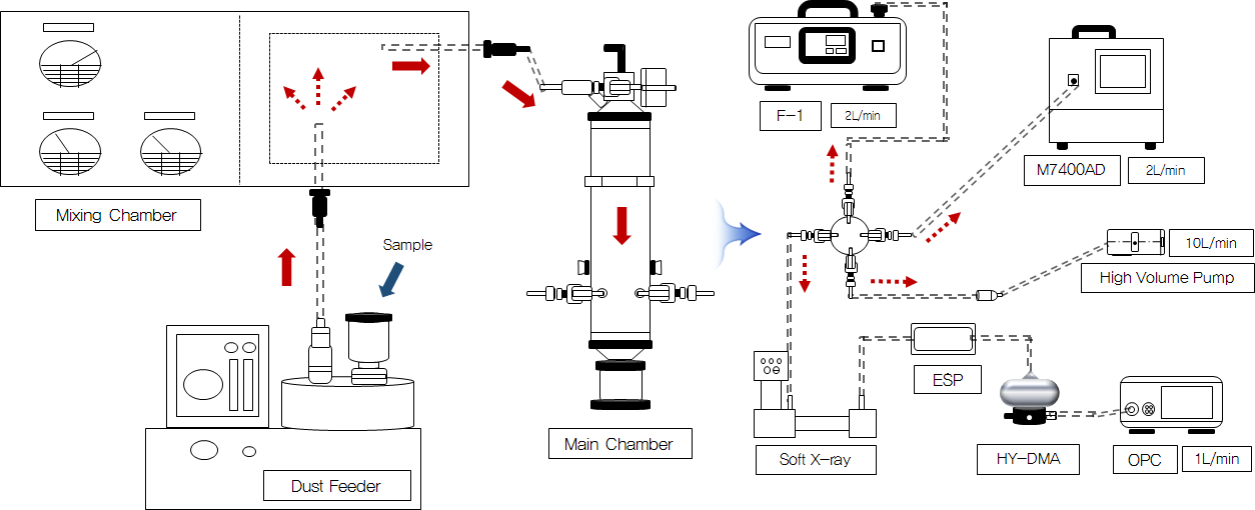
Schematic diagram comparing various asbestos measuring devices.

The test equipment for generating consistent fibrous matter was composed of three parts: (1) a dust feeder (Sibata, Japan) that supplied asbestos samples into the holes of the turn table where they were subsequently sucked in to the mixing chamber, (2) the mixing chamber where the samples were homogenously mixed, and (3) the main chamber where the measuring devices were connected to each port to collect the asbestos samples. Airborne asbestos fibers were collected using a KOFAM, an M7400AD, and an F-1, and the results were compared with those of the PCM method (Fig 1).

### 3. Instrument

**Fig 2:**
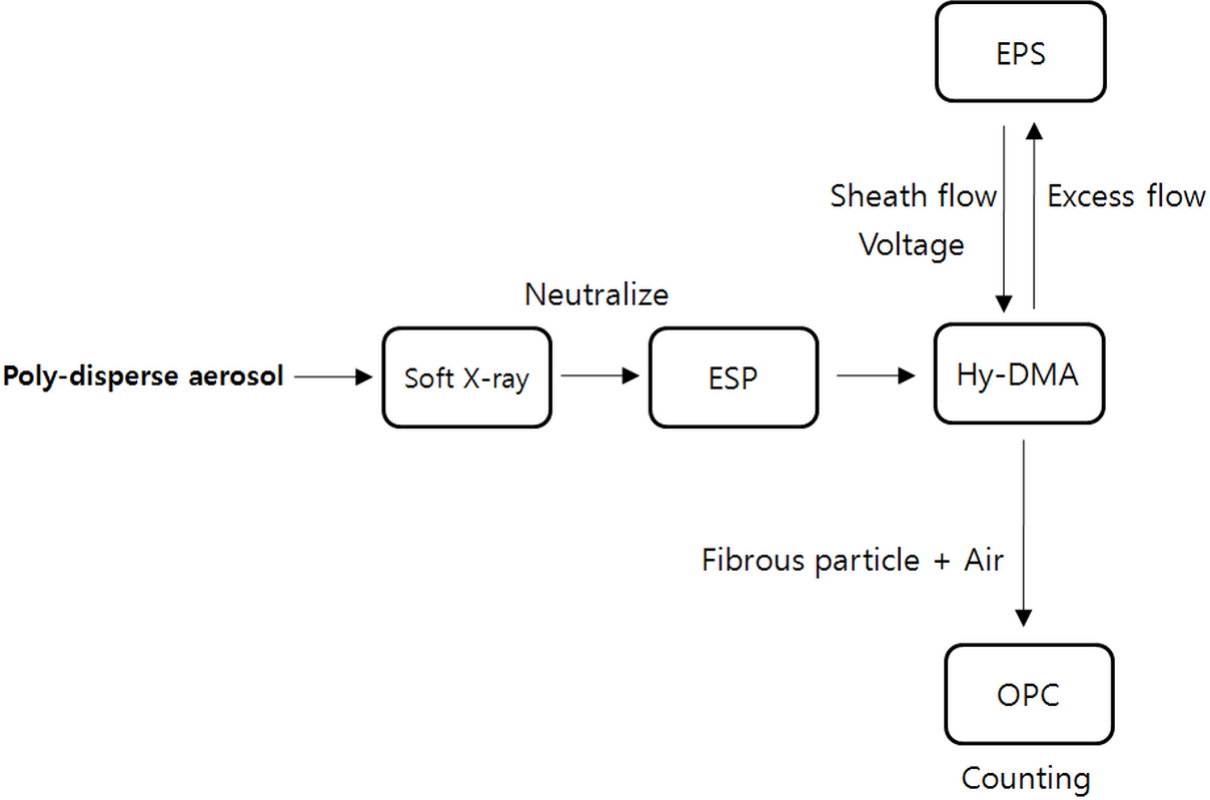
Flowchart of the components and processes comprising the KOFAM.

In order to analyze fibrous matter in real time, we developed a real-time measurement instrument by relying purely on Korean technology, and its patent applications have been filed. First, the soft X-ray charger (HCT, Korea) is used to bring the ion balance of irregularly charged particles in the sampled air to ±0V. However, some non-charged particles exist inside the soft x-ray charger. The electrode plates, which are charged with the positive and negative electrodes inside the electrostatic precipitator (ESP), are used to remove the charged matter in aerosols, and only the electroneutral particles are transferred to the HY-DMA. The air flows through an irregular electric field while passing through the HY-DMA, and a dipole moment takes place in the fibrous matter of the inflow air. While passing through, the air is affected by a regular electric field, which increases the mobility in accordance with the electrostatic forces of attraction between the fibrous matter and other matter, and subsequently the fibrous matter is separated from the rest. It is designed to measure the intensity of light scattered by the separated fibrous matter by using light scattering and to count only the fibrous matter whose length is 5μm or longer (Fig 2).

#### Soft X-ray charger

The soft X-ray charger is an instrument that brings the ion balance of irregularly charged particles in the sampled air to ±0V. The soft X-ray has a high ion generation density and a high electro-neutralization speed. It is easier to use than other instruments that utilize radioactive particles such as ^241^Am, ^85^Kr, and ^219^Po, generates no dust, and therefore does not require repair & maintenance. The charger is used to connect with the HY-DMA. It neutralizes the charged particles in the air and transfers them to the HY-DMA.

#### Electro-static precipitator (ESP)

The electrostatic precipitator removes the non-neutralized matter in aerosols from the soft X-ray charger and transfers only the electro-neutral matter to the HY-DMA. Inside the electrostatic precipitator, there are two electrode plates. One is charged with a positive electrode and the other is charged with a negative electrode, to remove the charged matter in aerosols. In this study, a voltage of 10 V was applied to the electrostatic precipitator.

#### HY-differential mobility analyzer (HY-DMA)

A DMA is an essential tool for aerosol researchers. Several different types of DMAs have been developed and are widely used. Generally, a DMA consists of two concentric cylindrical columns. With this configuration, a long column is required for large size particle generation or measurement. To overcome this problem, a toroidal type DMA was developed called an HY-DMA [13].

**Fig 3:**
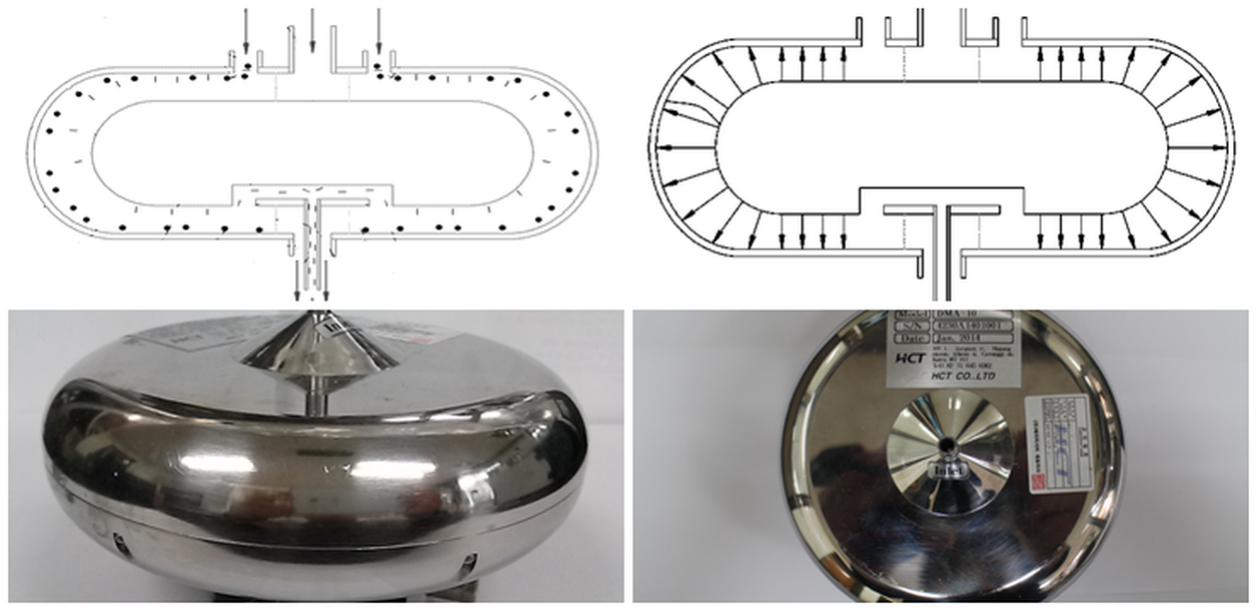
Schematic diagram and photo of an HY-DMA.

An HY-DMA is a device that separates fibrous matters from aerosols using a soft x-ray charger that removes any electrical charges on the particulate matter [13]. Therefore, only electro-neutral particles pass through the device. It consists of two parts. The first part has two electrodes that form a dipole moment around the fibrous matter by creating a non-uniform electric field between the electrodes. While creating this non-uniform electric field between the two electrodes, the second part generates a difference in the mobility of the fibrous matter around which dipole moment is created. When a voltage is applied to the HY-DMA through a voltage application device, an electric field is created between the inner and outer electrodes. The sampled air passes through the non-uniform electric field while travelling along the side of the HY-DMA. A dipole moment is generated around the fibrous matter, which is affected by the uniform electric field while moving towards the bottom of the HY-DMA. At this time, only fibers move towards the inner electrode because of the differences in mobility or electrostatic gravity between the fibers and other particulate matter, thereby separating them from the particulate matter (Fig 3).

#### Electrical particle sizer (EPS)

The electrical particle sizer (EPS) is an instrument connected to the HY-DMA. It applies the voltage required for polarizing the poly-disperse particles into mono-disperse particles. The sizer is capable of assigning voltage ranging from 10 to 7,000 V and capturing the desired sized particle (7–830 nm) as it sets different sizes for particles polarized at each voltage level.

#### Optical particle counter (OPC)

The optical particle counter (OPC) is an instrument that checks and counts the particles in the inflow air using light scattering and expresses them as numerical data. When the light is irradiated on particulate matter floating in the air, the light is scattered by the particulate matter. By measuring the intensity of the light scattered by a particle passing through a beam of laser or white light source, the counter identifies the size of the particle and counts it to identify the number of particles.

### 4. Recovery and accuracy of the KOFAM

We investigated different sheath-to-aerosol flow ratios to determine which would produce the internal flow required to optimally separate the fibers and yield a maximum concentration of mono-dispersed particles [17].

First, the flow was controlled to achieve a sheath-to-aerosol flow ratio of 10:1, which has previously been found to have the maximum DMA classification efficiency [18,19]. Subsequent tests were carried out at other ratios to assess their fibrous material separation capabilities.

To confirm the separation of particulate and fibrous materials inside the HY-DMA, they were compared before and after HY-DMA processing. Following confirmation, the optimum voltage for the separation of fibrous material was selected by applying voltages ranging from 10 to 7,000 V to the HY-DMA.

Three different concentrations, 0.01 f/cc (low level), 0.1 f/cc (medium level), and 0.5 f/cc (high level), of serpentine (chrysotile) and amphibole (amosite, tremolite, and actinolite) samples were prepared to perform the experiment three times in order to confirm the recovery rate by each type of asbestos. The recovery was evaluated by capturing the particles in the atmosphere and the particles that had passed through the HY-DMA. The recovery percentage was calculated using the formula shown in Eq (1):

$$\mathrm{Recovery}(\%)=\frac{C_{\mathrm{Hy-DMA}}}{C_{\mathrm{PCM}}}\times 100$$ (1)

$$\begin{array}{c} C_{\mathrm{HY-DMA}}:\;\mathrm{Airborne}\;\mathrm{fiber}\;\mathrm{concentration}\;\mathrm{captured}\;\mathrm{at}\;\mathrm{the}\;\mathrm{outlet}\;\mathrm{of}\;\mathrm{HY-DMA}\\ C_{\mathrm{PCM}}:\;\mathrm{Airborne}\;\mathrm{fiber}\;\mathrm{concentration}\;\mathrm{analyzed}\;\mathrm{by}\;\mathrm{PCM}\;\mathrm{method}(*\mathrm{NIOSH}\;7400’A’)] \end{array}$$

* The NIOSH 7400”A” method is the most widely used asbestos fiber analysis method currently in use, and analyzes asbestos fibers using a phase contrast microscope. Using the pump, it captures the air in the mixed cellulose ester (MCE) filter. The filter that extracts the sample is made transparent by an acetone vapor instrument, triacetin is firmly fixed (preprocessing), and then an analysis is performed using the phase contrast microscope. The fibrous matter whose length is 5μm or longer and whose length to diameter ratio is 3:1 or higher are counted using the Walton-Beckett graticule. The analysis results are expressed in fibers/cc (fibers per cubic centimeter of air).

### 5. Repeatability and accuracy evaluation

We examined the repeatability of the KOFAM by using asbestos samples of specifically varied concentrations (1 × 10−5–0.05% (w/w)). The experiment was repeated four times for a total of six different concentrations, and 24 samples were prepared for each asbestos type. A total of 44 samples were used in order to examine the differences in concentrations measured between the KOFAM and the PCM method. We also compared the coefficient of variation (CV) from our results against those of existing commercial devices, namely M7400AD and F-1. The coefficient of variation refers to a measure of the relative variability and is used to measure the degree of spread of result values relative to one another. The coefficient of variation is a statistical method mainly used to compare two or more data sets whose units are different, or whose central locations are widely different. A calculated coefficient of variation closer to 0 means that it is clustered more around the mean and the degree of spread is lower. The coefficient of variation is calculated using the following equation (Eq 2):

$$\mathrm{CV(\%)}=\frac{\sigma }{x}\times 100$$ (2)

$$\begin{array}{c} \sigma \;:\;\mathrm{Standard}\;\mathrm{Deviation}\;\mathrm{of}\;\mathrm{Result}\;\mathrm{data}\\ x\;:\;\mathrm{MEAN}\;\mathrm{of}\;\mathrm{Result}\;\mathrm{data} \end{array}$$

The accuracy of the KOFAM was assessed by using unknown concentration samples. The test was performed 146 times for each sample, for a total of 292 tests. A high volume pump with a cowl containing an MCE filter (0.8 μm pore-size, 25 mm diameter) was connected to a port of the main chamber and collected samples at a flow rate of 10 L/min for 40 min. The flow rate of the KOFAM was set to 1 L/min and samples were collected for 40 min. Samples were also collected using an M7400AD and an F-1 at a flow rate of 2 L/min for 40 min in order to comparatively assess airborne asbestos concentrations.

### 6. Analytical method

The sampled filters were analyzed according to the NIOSH 7400 ‘A’ counting method [4]. At most, 100 fibers or 100 fields of a preprocessed filter were observed using a phase contrast microscope (Olympus, Japan) with WB graticule. The error of each method compared to that of PCM was obtained by calculating the relative difference (R_D_), as shown in Eq (3):

$$R_{D}=\frac{ABS(C_{KOFAM}-C_{PCM})}{C_{PCM}}$$ (3)

$$\begin{array}{c} \mathrm{ABS}:\;\mathrm{Absolute}\;\mathrm{value}\\ C_{\mathrm{KOFAM}}:\;\mathrm{Airborne}\;\mathrm{fiber}\;\mathrm{concentration}\;\mathrm{measured}\;\mathrm{by}\;\mathrm{the}\;\mathrm{KOFAM}\\ C_{\mathrm{PCM}}:\;\mathrm{Airborne}\;\mathrm{fiber}\;\mathrm{concentration}\;\mathrm{analyzed}\;\mathrm{by}\;\mathrm{the}\;\mathrm{PCM}\;\mathrm{method} \end{array}$$

### 7. Statistical analysis

All the experiments were repeated four times, and descriptive statistics on the fiber concentrations were calculated; median, minimum, and maximum values. Relative difference (R_D_) was used to verify the difference between the results of the PCM and KOFAM methods. Linear regression and the least square method were used to calculate the relationship and coefficient of determination (R^2^) between the PCM and KOFAM. The conformity between the two measured values was assessed utilizing a Bland and Altman plot.

## Results

### 1. General characteristics

**Fig 4:**
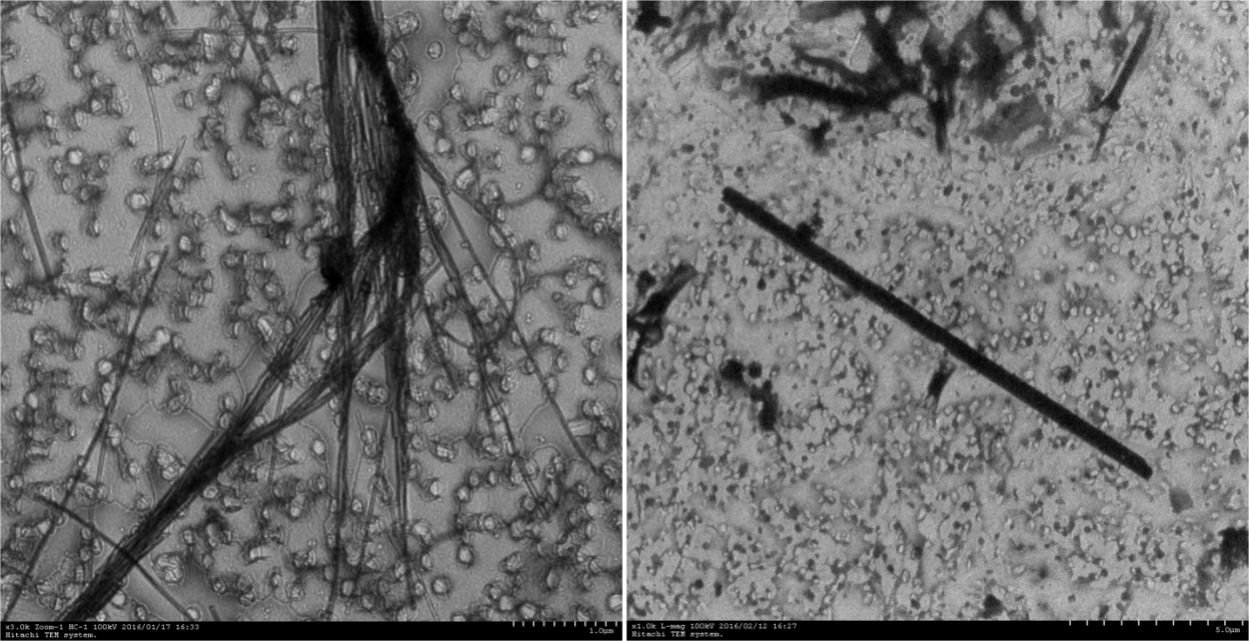
Transmission electron microphotograph of serpentine and amphibole asbestos (samples were respirable reference materials from RTI).

The general characteristics of the asbestos used in this study are described in Table 1. Regarding the morphological characteristics, the serpentine type (chrysotile) had a curly configuration, and the amphibole type (amosite, tremolite asbestos, and actinolite asbestos) had a straight needle shape. The aspect ratio of chrysotile exceeded 28:1, and that of the amphibole type was approximately 18:1 (Table 1 and Fig 4).

**Table 1:** General characteristics of the asbestos used in the experiment.

Asbestos	Length (μm)	Width (μm)	Aspect ratio	Morphology
Serpentine (chrysotile)	7.99±10.47	0.23±0.18	28.0±26.6	Straight to wavy fibers and bundles, kink structures, splayed bundle ends
Amphibole (amosite, tremolite, actinolite)	12.23±9.49	0.70±0.39	18.7±14.2	Straight, rigid bundles

### 2. Fiber generation

To examine the homogeneity of the particle measurement sampling ports on the test device, we conducted several experiments using the two types of samples. The results showed that the coefficient of variation (CV) of each port was between 0.52 and 2.15% relative to the asbestos content. In other words, the airborne asbestos samples were dispersed homogenously within the chamber, and the concentrations measured at each of the six ports were no different from each other.

### 3. Separation of fibers from particulate matter

The existence of fibrous materials was monitored by changing the sheath and aerosol flow rates so that the HY-DMA had optimal separation capacity, beginning with a ratio of 10:1, where the DMA had maximum classification efficiency, and gradually working down to lower ratios. Fibrous material was not observed when at ratios from 10:1 to 2:1, but was observed at ratios less than 2:1. However, particulate matter was also detected mixed in amongst the separated fibrous materials. A range of ratios from 1:1 to 2:1 was specifically monitored at intervals of 0.1 L in order to identify when fibrous materials were most effectively separated, and it was found that the highest separation efficiency occurred at a ratio of 1.6:1 (Table 2).

**Table 2:** Separation capacity of fibrous and particulate materials by flow rates.

			sheath flow: aerosol flow (L)							
		10:1	5:1	5:0.5	2:1	2:0.2	1.7:1	1.6:1	1.5:1	1:1
Serpentine	excess	△	△	△	△	△	△	△	△	△
	outlet	-	-	-	-	-	△	△	△	△
Amphibole	excess	△	△	△	△	△	△	△	△	△
	outlet	-	-	-	-	-	△	△	△	△

-: fibrous materials were not observed, △: a mixture of fibrous and particulate materials was observed, outlet: only fibrous materials that passed through was observed, excess: where fibrous and particulate materials that did not pass through were observed

**Fig 5:**
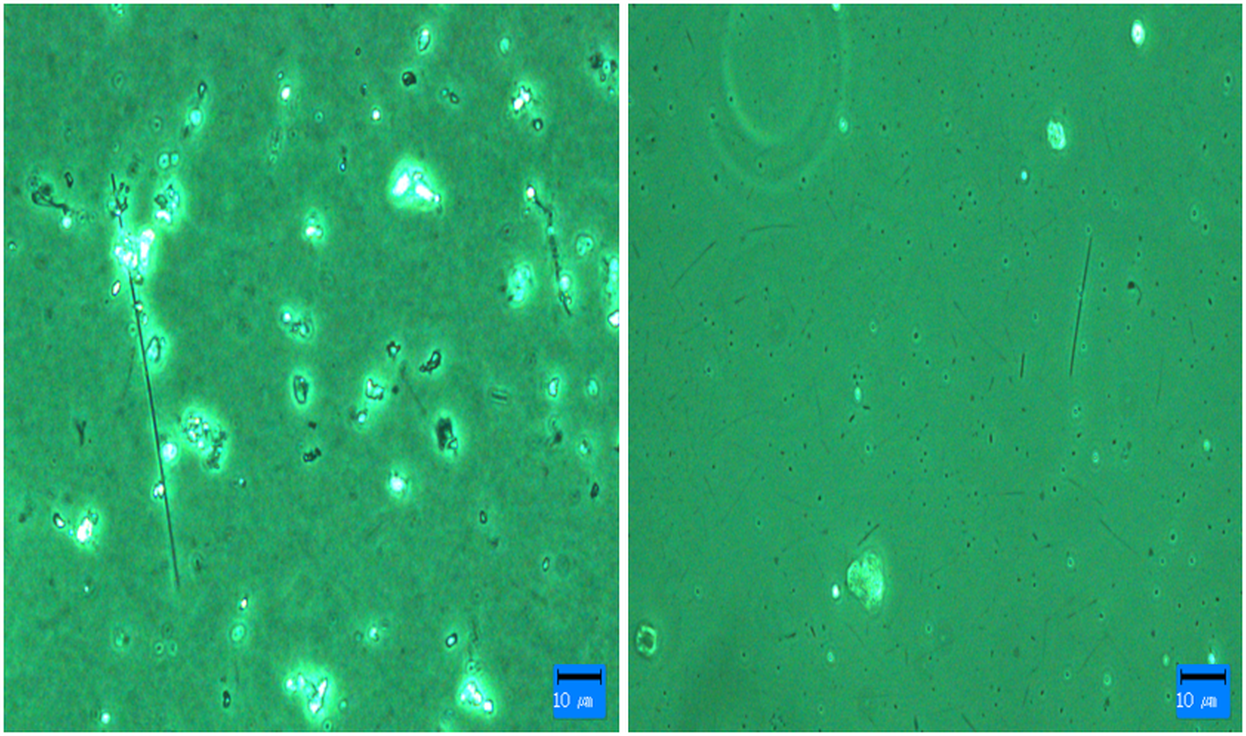
Separation of fibers from particulate matter with the HY-DMA. Left: Filter prepared for PCM (400×) analysis (air not passed through HY-DMA). Right: Filter prepared for PCM (400×) analysis (air passed through HY-DMA and particulate matter removed).

The ability of the HY-DMA to effectively separate fibers from particulate matter was tested by using PCM to analyze filters for air that and had not been passed through the HY-DMA (Fig 5) with sheath and aerosol flows of 1.6 L/min and 1 L/min, respectively. Mixed fibrous and particulate matter was observed on the filters for air that did not pass through the HY-DMA. In contrast, only fibrous matter was found on the filters for air that passed through the HY-DMA. Thus, it was confirmed that the HY-DMA is capable of separating fibers from particulate matter.

### 4. HY-DMA voltage selection according to recovery and length distribution

**Fig 6:**
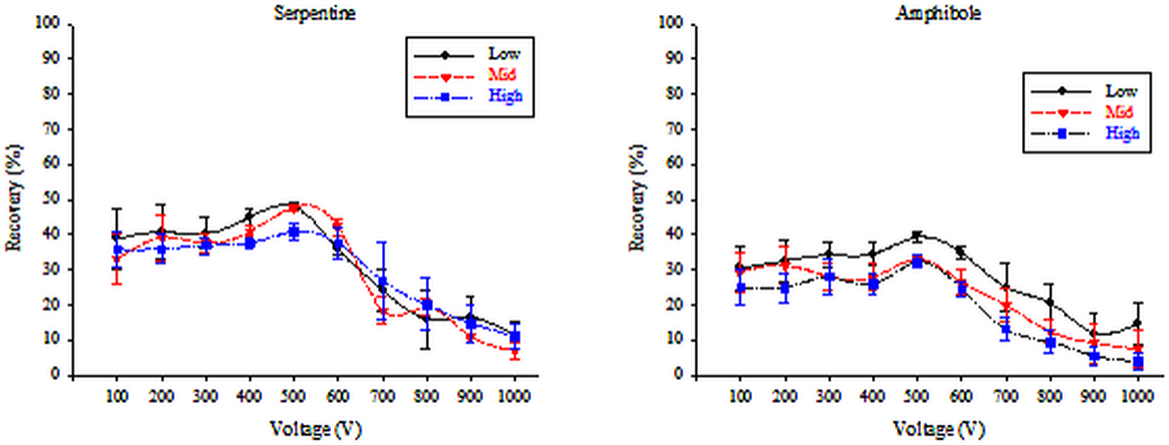
HY-DMA recovery according to asbestos type, concentration, and applied voltage.

To determine the optimum voltage required to maximize the separation capacity of the HY-DMA, voltages ranging between 10 and 7,000 V were tested. The sample recovery was found to be the highest at 500 V for all the asbestos samples tested; absolute recovery rates depended on the types of asbestos as well as the concentrations. From the results of the recovery observation, the recovery tended to increase as the applied voltage increased starting at 100 V. The highest recovery was observed at 500 V. The recovery by concentration was as follows. In the case of the serpentine type, the highest recovery was 48.0±1.0% at the low concentration level (0.01 f/cc), and was 47.8±0.8% for both the medium (0.1 f/cc) and high (0.5 f/cc) concentration levels. In the case of the amphibole type, the maximum recovery rate was 47.8±0.8% for the low concentration level, 33.1±0.8% for the medium concentration level, and 33.1±0.8% for the high concentration level. The recovery rate started to decrease as the voltage became greater or equal to 600 V (Fig 6).

**Fig 7:**
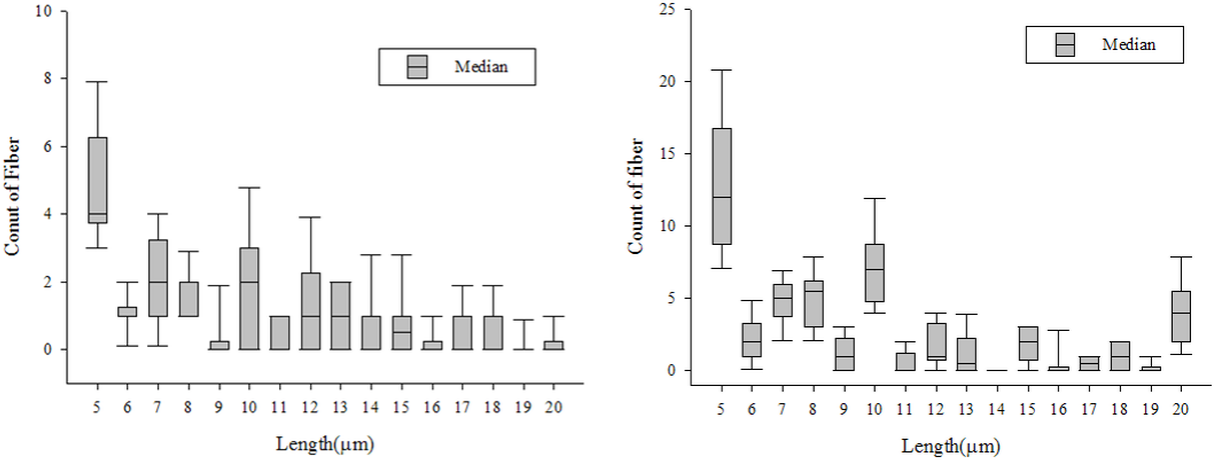
Length distribution of asbestos type generated(serpentine, amphibole type).

**Fig 8:**
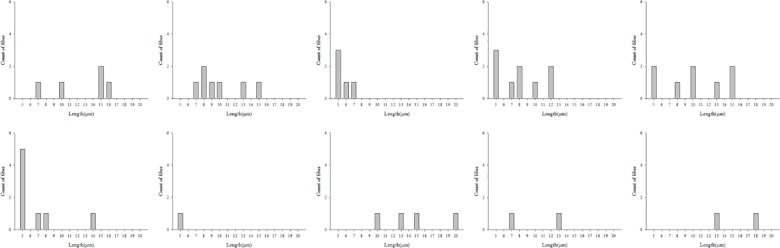
Length distribution according to applied voltage(serpentine type; 100–1,000V).

**Fig 9:**
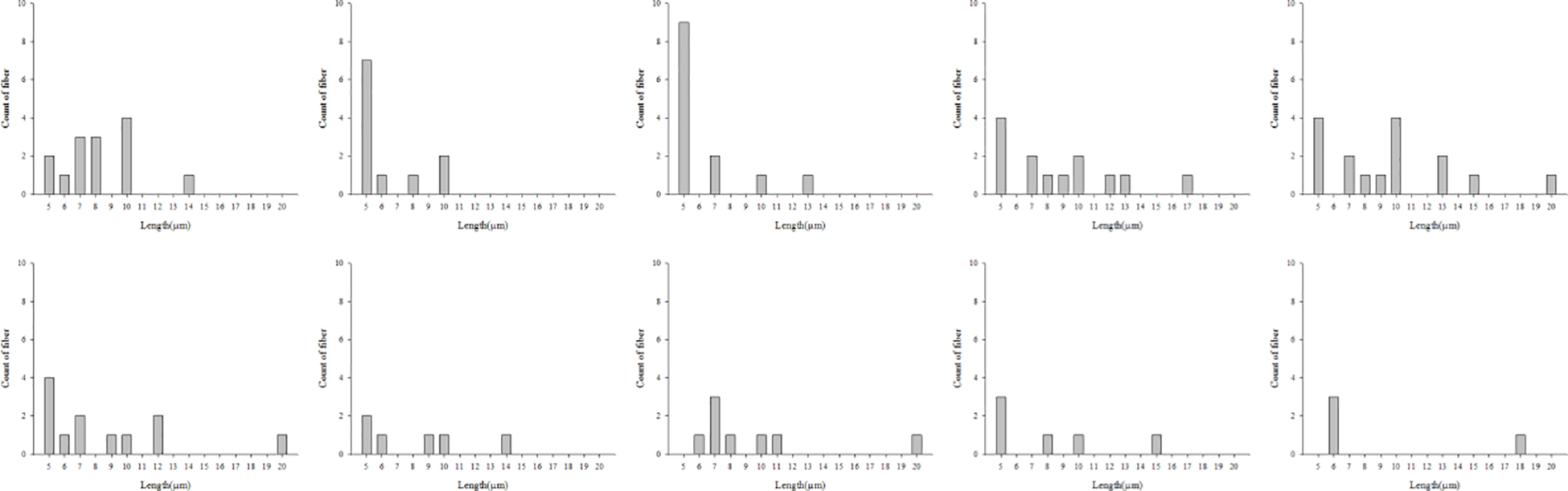
Length distribution according to applied voltage(amphibole type; 100–1,000V).

Moreover, the length distribution of asbestos fibers separated from HY-DMA have been examined. They were mainly short fibers (5–10 μm), but the length distribution caused by the change in voltage was not even and no difference between asbestos types was found (Figs 7–9).

### 5. Establishment of specific correction factors

We verified the correlations between the concentrations measured from reference materials and those obtained by the PCM method. However, since 50% of the input particles were lost due to particle loss functions inside the DMA, we applied a specific correction factor on the 50% recovery, which was acquired from the KOFAM. Therefore, a correction factor of 2.98 obtained from Eq (4) was applied:

$$\mathrm{KOFAM}\;(f/\mathrm{cc})=\frac{F_{5\mu m}}{40\min\times 1,000}\times **k$$ (4)

$$\begin{array}{c} F_{5\mu m}:\;\mathrm{Sum}\;\mathrm{of}\;\mathrm{total}\;\mathrm{fibers}\;\mathrm{longer}\;\mathrm{than}\;5\mu m\\ k:\;\mathrm{factor}\;(2.98) \end{array}$$

According to NIOSH 7400 “A,” which is currently used as an analysis method, a variation of -49% to 213% occurs when 100 fibers are counted in a single sample. In addition, the same concentration cannot be gained repeatedly from one sample due to reasons such as differences in preprocessing and analysis techniques of the analyst, fatigue of the eyes, and different phase contrast microscope models. In this regard, this study applied an adjusting coefficient to the concentrations measured by KOFAM to reduce the level of error in analysis and brought them within the variation analyzed by the phase contrast microscope [4].

### 6. Comparison of concentrations measured by instrument

**Fig 10:**
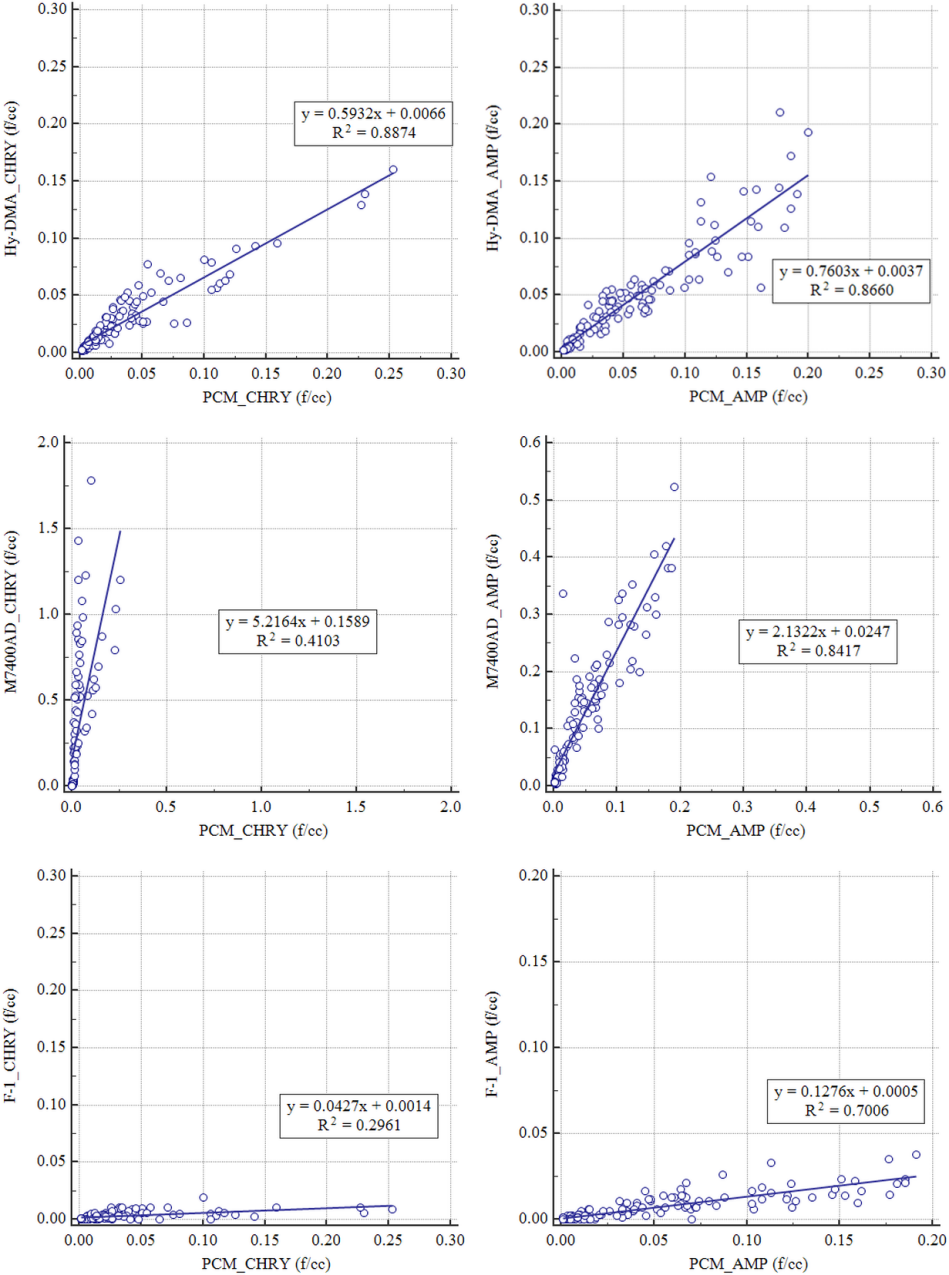
Correlation plots of PCM versus other methods for unknown concentration samples.

The concentrations measured by the KOFAM, M7400AD, and F-1 were compared to the concentration measured by PCM as the gold standard. When the KOFAM, M7400AD, and F-1 were compared, the results of the KOFAM were more accurate than the M7400AD and F-1 fiber monitors. The results of the linear regression analysis between the concentrations measured with the KOFAM and PCM showed high correlation; the R^2^ value of serpentine was 0.89 and that of amphibole was 0.87, whereas the R^2^ serpentine and amphibole values for the M7400AD and PCM correlation were 0.41 and 0.84 for amphibole, respectively, and those for F-1 and PCM were 0.30 and 0.70, respectively (Fig 10).

We evaluated the accuracy by dividing the PCM versus KOFAM, M7400AD, and F-1 results into each level. The KOFAM showed the highest accuracy compared to other methods at a value of 72.9%. The comparison results for each level showed that the accuracy of serpentine types and amphibole types were as follows: 62.1% and 76.6% for level 1; 73.7% and 75.9% for level 2; 74.1% and 84.3% for level 3; 66.4% and 75.1% for level 4; and 58.6% and 77.0% for level 5, respectively (Table 3).

**Table 3:** Summary of relative errors of KOFAM, M7400AD, and F-1 on unknown concentration samples.

						(f/cc, Median(min-max))	
Asbestos	Level*	n	Temp.† (°C)	Humidity† (%)	R_D_-KOFAM**	R_D_-M7400AD**	R_D_-F-1**
Serpentine	1	31	22.7±1.3	30.5±3.1	32.9(0.5–72.8)	216.4(8.3–568.1)	75.6(41.2–78.7)
	2	27	23.1±1.0	34.1±2.2	26.3(0.5–48.0)	317.2(1.0–2182.8)	81.8(50.5–89.2)
	3	63	21.7±0.9	36.1±1.9	25.9(0.5–67.2)	1139.9(4.0–4145.9)	86.8(51.6–96.3)
	4	13	22.4±1.7	34.4±2.7	33.6(2.8–69.0)	997.9(27.0–2034.4)	86.8(80.5–94.8)
	5	12	22.1±1.8	33.9±3.1	41.4(25.4–49.0)	365.2(33.0–447.4)	95.9(93.3–98.6)
		146			31.2(0.5–72.8)	357.2(1.0–4145.9)	86.4(41.2–98.6)
Amphibole	1	23	23.1±1.7	37.6±4.1	23.4(7.1–47.8)	248.9(8.5–3664.7)	51.2(16.7–100.0)
	2	20	22.5±1.3	33.9±2.6	24.1(0.4–47.7)	240.2(92.2–429.0)	82.8(65.5–89.2)
	3	48	21.4±0.7	35.1±4.6	15.7(0.6–68.2)	220.4(21.9–2217.2)	84.4(55.5–97.0)
	4	27	21.0±0.8	37.3±2.3	24.9(0.8–48.9)	148.6(41.9–244.1)	86.5(68.1–92.7)
	5	28	22.1±1.1	36.9±4.3	23.0(2.1–64.7)	102.8(47.9–215.8)	88.7(70.6–94.5)
		146			21.8(0.4–68.2)	176.6(8.5–3664.7)	85.8(16.7–100.0)

*level 1: 0.001–0.005 f/cc; level 2: 0.005–0.010 f/cc; level 3: 0.010–0.050 f/cc; level 4: 0.050–0.100 f/cc; level 5: more than 0.100 f/cc

†Temp. = Temperature, Humidity = Relative humidity

**R_D_ = Relative difference

### 7. Precision assessment

The CVs of the concentration values obtained from serpentine and amphibole samples were examined to assess precision. In general, lower CVs occurred as the sample content increased. We confirmed that the KOFAM had the most similar CV to PCM as evidenced by the PCM, KOFAM, M7400AD, and F-1 values of 21.9, 22.5, 32.4, and 88.8, respectively (Table 4).

**Table 4:** Precision assessment of the KOFAM, M7400AD, and F-1.

Asbestos	Content (%)	n	CV_PCM_	CV_KOFAM_	CV_M7400AD_	CV_F-1_
Serpentine	0.00001	4	26.1	21.8	55.2	115.5
	0.00005	4	15.2	38.8	41.7	41.3
	0.0001	4	34.7	28.9	57.9	91.1
	0.001	4	30.1	26.6	59.3	126.9
	0.01	4	10.9	15.8	26.2	75.7
	0.05	4	12.7	18.7	8.6	23.4
		24	21.6	25.1	41.5	79.0
Amphibole	0.00001	4	18.9	29.5	10.3	115.5
	0.00005	4	16.1	22.0	18.7	66.7
	0.0001	4	20.8	15.4	26.0	119.3
	0.001	4	10.1	7.2	9.7	157.1
	0.01	4	45.7	23.3	42.5	44.8
	0.05	4	-	-	-	-
		20	22.3	19.5	21.5	100.7
Average			21.9	22.5	32.4	88.8

### 8. Comparison of measurement methods using a Bland & Altman plot

**Fig 11:**
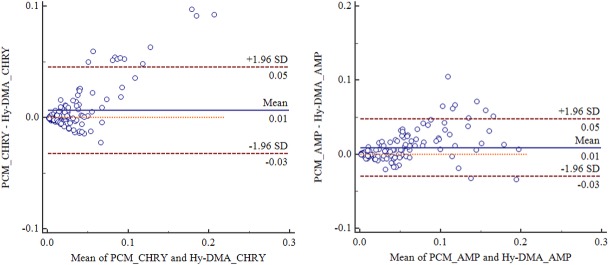
Comparison of KOFAM and PCM methods using a Bland & Altman plot.

To assess the conformity of measured values, we conducted a Bland and Altman analysis. For the serpentine type, the average difference between the concentration values measured with the KOFAM and PCM was 0.006 (95% CI; -0.038–0.045), and the standard deviation was 0.0198. For the amphibole type, the average difference between the concentration values was 0.009 (95% CI; -0.030–0.047), and the standard deviation was 0.0197. The majority of concentration values were within ± 2 SD (Fig 11).

## Discussion

Setting the sheath-to-aerosol flow ratio to 10:1 was previously established as the ratio that provides the most stable flow and yields the maximum classification efficiency of nano-level particles in the DMA [18,19]. However, because the objectives of these studies differed from that of this study, the experimental conditions for obtaining the maximum classification efficiency of particles needed to be determined for this study.

Based off of the work of Kim et al. (2014) who showed that the concentration of extracted mono-disperse particles increases after classification, even though the maximum classification efficiency and the degree of size uniformity (mono-dispersity) decrease with increasing poly-dispersed particle flow rate, an adequate sheath-to-aerosol flow ratio inside the HY-DMA appropriate for the objective of this study was identified [17]. Fibrous material was not detected at the outlet of the HY-DMA at ratios greater than 2:1, but a mixture of fibrous and particulate material was observed at ratios less than 2:1. By monitoring the specific range of 1:1 to 2:1 at 0.1 L intervals, we confirmed that separation was most efficient at a ratio of 1.6:1.

Differences in electrostatic mobility, which is the basic principle upon which the HY-DMA operates, were used to separate fibers from the particulate matter. In this study, separation efficiency was assessed for a voltage range of 10 to 7,000 V. Because the separation efficiency of the HY-DMA dropped when a voltage over 1,000 V was applied, it was excluded from the results (Fig 9). Based on recovery values of the HY-DMA for various voltages, the optimum voltage was found to be 500 V for all four asbestos samples.

We found that the recovery values by concentration, for the serpentine types, were 48.0±1.0% for the low level (0.01 f/cc), 47.8±0.8% for the medium level (0.1 f/cc), and 40.9±2.2% for the high level (0.5 f/cc). Moreover, the recovery values by concentration for the amphibole types were 39.3±1.3% for the low level, 33.1±0.8% for the medium level, and 32.3±1.8% for the high level. The reason for low recovery was that 50% of the input particles were lost due to the particle loss functions inside the DMA [20]; therefore, 100% separation efficiency was reached when the real recovery of the HY-DMA was 50%.

In Griffith’s study, a mobility spectrometer-based device was developed to separate fibrous and particulate matters, showing that it was more efficient to separate fibers whose aspect ratios were 20:1 than fibers whose aspect ratios were 10:1 [21]. Further, through actinolite asbestos-based research, Yu found that the higher the aspect ratio, the more efficient electrification was [12]. Furthermore, Han et al. (1994) showed that charged fibers with higher aspect ratios could be electrified more efficiently [22]. Similarly, Ku et al. (2011) reported that CNT with a higher height-to-width aspect ratio was more efficient in electrification [7].

High separation efficiency was shown for serpentine, which has a relatively large aspect ratio compared to the other asbestos samples used in this study. Accordingly, we believe that a high recovery was obtained.

In this study, we employed OPC as a means of measuring particulate matter. This was because previous studies showed that OPC could detect fibrous materials when only fibrous materials existed [14,15], and both PCM and OPC had the same resolution of 0.25 μm; therefore, we concluded that comparison with PCM method was appropriate [23].

A specific constant (*k*) was introduced to solve the problem of underestimation because compared with PCM, the maximum recovery of KOFAM was less than 50% due to the DMA characteristics. Through comparison of PCM and the KOFAM in a total of 292 experiments, a correction factor of 2.89 was obtained (Table 3).

We counted fibers using an OPC after applying a correction factor (*k*) to the fibers separated by the HY-DMA method (Table 3 and Table 4). A comparative analysis between the KOFAM and the PCM method indicated that the relative differences of the serpentine type and amphibole type were 31.2 and 21.8, respectively. Lower relative difference values implied more accurate measurements. Regarding the CV analysis, the CV of the KOFAM was approximately 22.5%, and those of the M7400AD, F-1, and PCM were 32.4%, 88.8%, and 21.9% respectively. Kauffer et al. (2003) presented findings showing that the standard deviation of concentrations measured with the HY-DMA was less than 16% [24]. Results presented by Hiromoto et al. (1997) were similar to those of this study in that the standard deviation of the PCM method was approximately 20% and the standard deviation of the asbestos real-time monitor (ARM) was approximately 16% [25].

Phanprasit et al. (1988) compared a fibrous aerosol monitor (FAM) and PCM. When 33 samples were classified into low-concentration (<1.0 f/cc) and high-concentration (>1.0 f/cc) categories and 4 μm or larger sized fibrous matter was counted, the correlation coefficients (r) at low and high concentrations were 0.70 and 0.79, respectively, and the r value of all samples was 0.92. In contrast, when 5 μm or larger sized fibrous matter was counted, the r value at the low and high concentrations were 0.72 and 0.87, respectively, and the r value of all samples was 0.959 [26]. In a study by Hiromoto et al. (1997), the concentrations of amosite measured by an ARM device and the PCM method were compared. Their results showed the calibration coefficient (α) to be 26.10±0.36 and the r value to be 0.9810 [25]. To verify the correlation between two devices in this study, we conducted a linear regression analysis and calculated the R^2^ value, instead of the correlation coefficient. The R^2^ values of serpentine and amphibole were 0.89 and 0.87, respectively, which indicates that there were significant correlations (Fig 10).

To assess the conformity of concentrations analyzed by the KOFAM and PCM, we carried out a Bland and Altman plot. This type of plot is generally used to verify the accuracy of a new method relative to existing ones by analyzing the difference between the averages of the methods [27]. The smaller the average distribution of differences (MEAN ± 2 SD), the higher the conformity is between the two methods. If the distribution is sufficiently small, then the new method can replace the old one. The more closely two measured values conform to each other, the more the Y value approaches zero. As such, we were able to ensure conformity between the two devices by the fact that the majority of the average difference distributions of the concentrations measured by each device were within ± 2 SD. Here, the concentration measured by the KOFAM was lower than the concentration measured by PCM (Fig 11).

Similar to these results, Kauffer et al. (2000) showed that the concentration of chrysotile fibers generated in a chamber measured with an FM-7400 was significantly lower than that measured by PCM [28]. Table 4 illustrates the phenomenon in which the trend of the separation efficiency of the HY-DMA was reflected in the OPC measurements. Our study suggests that if fibrous and particulate matters are not well separated due to the low separation efficiency of the instrument, then the result of measurement by OPC also declines. Therefore, we believe that high separation efficiency of the HY-DMA is the key to the real-time detection of fibers using the HY-DMA/OPC combination.

Lastly, we think that, similar to the results of relative differences, the relative differences between the rates of the KOFAM and PCM could be reduced with increasing recovery of the HY-DMA. In addition, controlling neutralization capacity as well as minimizing irregularly charged particles in the HY-DMA should be investigated to reduce these differences. We assume future measurements will be more accurate if there are improvements to the HY-DMA in follow-up studies. For example, enlarging the diameter of the HY-DMA would facilitate the separation of high concentration fibrous matter by increasing the contact time of sampled air within the device. Moreover, since this study was conducted exclusively in a laboratory, unexpected factors under real field conditions need to be considered in the future. For example, temperature and humidity, non-uniform particulate matter, and wind may interfere with measurements. Thus, field assessments should be conducted in the future to assess the applicability of the device.

## Conclusions

We found no statistically significant differences of asbestos concentrations between the KOFAM and PCM analyses. Thus, the KOFAM device has been shown to be an effective substitute for the PCM method, and is capable of detecting fibers in real time. Therefore, it may be used as a measurement device that can continuously monitor asbestos concentration in real time. We believe that the KOFAM can be applied to monitor environmental release of asbestos fibers from abatement sites and ambient atmosphere.

## Supporting information

S1 Table General characteristics of the asbestos used in the experiment. (XLSX)

S2 Table Separation capacity of fibrous and particulate materials by flow rates. (XLSX)

S3 Table HY-DMA recovery according to asbestos type, concentration, and applied voltage. (XLSX)

S4 Table Length distribution of asbestos type generated(serpentine, amphibole type). (XLSX)

S5 Table Length distribution according to applied voltage(serpentine type; 100–1,000V). (XLSX)

S6 Table Length distribution according to applied voltage(amphibole type; 100–1,000V). (XLSX)

S7 Table Summary of relative errors of KOFAM, M7400AD, and F-1 on unknown concentration samples. (XLSX)

S8 Table Precision assessment of the KOFAM, M7400AD, and F-1. (XLSX)

S9 Table Comparison of KOFAM and PCM methods using a Bland & Altman plot. (XLSX)
